# Microglia Morphology in the Developing Primate Amygdala and Effects of Early Life Stress

**DOI:** 10.1523/ENEURO.0466-24.2024

**Published:** 2025-01-10

**Authors:** Dennisha P. King, Miral Abdalaziz, Ania K. Majewska, Judy L. Cameron, Julie L. Fudge

**Affiliations:** ^1^Department of Neuroscience, University of Rochester Medical Center, Rochester, New York 14642; ^2^ Department of Psychiatry, University of Pittsburgh, Pittsburgh, Pennsylvania 15213; ^3^Department of Psychiatry, University of Rochester Medical Center, Rochester, New York 14642

**Keywords:** adolescent, infant, maternal separation, paralaminar nucleus, stress

## Abstract

A unique pool of immature glutamatergic neurons in the primate amygdala, known as the paralaminar nucleus (PL), are maturing between infancy and adolescence. The PL is a potential substrate for the steep growth curve of amygdala volume during this developmental period. A microglial component is also embedded among the PL neurons and likely supports local neuronal maturation and emerging synaptogenesis. Microglia may alter neuronal growth following environmental perturbations such as stress. Using multiple measures in rhesus macaques, we found that microglia in the infant primate PL had relatively large somas and a small arbor size. In contrast, microglia in the adolescent PL had a smaller soma and a larger dendritic arbor. We then examined microglial morphology in the PL after a novel maternal separation protocol, to examine the effects of early life stress. After maternal separation, the microglia had increased soma size, arbor size, and complexity. Surprisingly, strong effects were seen not only in the infant PL, but also in the adolescent PL from subjects who had experienced the separation many years earlier. We conclude that under normal maternal-rearing conditions, PL microglia morphology tracks PL neuronal growth, progressing to a more “mature” phenotype by adolescence. Maternal separation has long-lasting effects on microglia, altering their normal developmental trajectory, and resulting in a “hyper-ramified” phenotype that persists for years. We speculate that these changes have consequences for neuronal development in young primates.

## Significance Statement

The paralaminar (PL) nucleus of the amygdala is an important source of plasticity, due to its unique repository of immature glutamatergic neurons. In rhesus macaques, similar to human, PL immature neurons mature between birth and adolescence. This maturation process is likely supported by synaptogenesis, which requires microglia. Between infancy and adolescence in macaques, PL microglia became denser and shifted to a “ramified” phenotype, consistent with increased synaptic pruning functions. Early life stress in the form of maternal separation, however, blunted this normal trajectory, leading to a persistent “hyper-ramified” microglial phenotype. We speculate that microglia hyper-ramification aligns with “para-inflammatory” concepts of stress and may alter PL neuronal maturation and synapse formation in young animals.

## Introduction

During infancy, human and nonhuman primates solidify a permanent foundation for their cognitive, social, and emotional health. One structure critical for social and emotional development is the amygdala (Bachevalier et al., 1999; Sanchez et al., 2001; Amaral et al., 2003; Gee et al., 2013; Tottenham, 2014), which detects and processes salient sensory cues in awake human and nonhuman primates (Wang et al., 2014; Wang et al., 2017; Grabenhorst et al., 2019; Morrow et al., 2020). Lesions and environmental factors can impact the maturation of the amygdala resulting in life-long consequences for affective behavior (Schumann et al., 2011; Payne and Bachevalier, 2019).

The amygdala volumetrically expands between infancy and adolescence in both monkeys and humans (Giedd et al., 1996; Uematsu et al., 2012; Hu et al., 2013; Goddings et al., 2014; Schumann et al., 2019). Despite neuroimaging evidence for volumetric growth of the amygdala, few studies have examined the cellular basis for this expansion. Prior studies in primates as well as in rodents indicate that the amygdala's mature neuron numbers increase between birth and adolescence. In humans, dendritic complexity also increases (Rubinow and Juraska, 2009; Weir et al., 2018; Avino et al., 2018; Sorrells et al., 2019; McHale-Matthews et al., 2023).

A potential source of increasing numbers of mature neurons in the amygdala during early life is the paralaminar nucleus (PL). The PL is a repository of immature glutamatergic neurons that persist postnatally. Between infancy and adolescence, the ratio of mature to immature neurons in the PL increases, suggesting active maturation (Avino et al., 2018; Sorrells et al., 2019; Chareyron et al., 2021; McHale-Matthews et al., 2023). In humans, other amygdala nuclei also gain mature neurons in the first decades of life (Avino et al., 2018), leading to the idea that neuronal maturation, and possibly migration, from the PL, is a potential mechanism for cellular expansion not only in the PL but also other amygdala nuclei.

One major question is: what neural processes support PL cellular maturation between infancy and adolescence? Emergent excitatory activity from afferent terminals is required for postsynaptic neuron development, stimulating the growth of dendritic spines, elaboration of dendritic arbors, and synapse formation (Engert and Bonhoeffer, 1999; Harris, 1999; Evers et al., 2006; Elston and Fujita, 2014; Oga et al., 2017). Thus, excitatory inputs that arrive in the amygdala postnatally may also shape PL neuron maturation (Cressman et al., 2010; Arruda-Carvalho et al., 2017). Environmental influences such as sensory changes and stress can therefore influence spinogenesis and synaptogenesis in the developing brain, presumably through effects on this process (Vyas et al., 2002; Mitra et al., 2005; Roozendaal et al., 2009; Block et al., 2022).

Microglia are immune cells that support neuronal migration (Xavier et al., 2015) and mediate neural network assembly in the developing brain through synaptic remodeling (Paolicelli et al., 2011; Hammond et al., 2018; Whitelaw et al., 2023). Microglial phagocytosis of weak synapses is considered essential during circuit formation (synaptogenesis) because it maintains and strengthens the remaining synapses, thereby enhancing neural transmission (Schafer et al., 2012; Zhan et al., 2014; Whitelaw, 2018). For example, classic studies of light deprivation in juvenile mice show increased microglial engulfment of weakened synaptic elements including synaptic clefts, resulting in repatterning of the visual field (Tremblay et al., 2010; Tremblay and Majewska, 2019). Despite its high degree of neuronal plasticity, microglia in the PL have not been previously characterized in a macaque.

In view of the extensive literature confirming that early environmental influences represent a major risk factor for the subsequent development of several psychopathologies (Kessler et al., 2003; Cameron et al., 2017; Catale et al., 2020), and the important role of microglia in establishing circuitry (Wei et al., 2015; Delpech et al., 2016), we asked whether developmental adversity in the form of maternal separation would alter microglial morphology, a potential sign of altered synaptogenesis. Using classic morphologic criteria, we investigated both normal PL microglial development, as well as the effects of modified maternal separation in infancy and adolescence.

## Materials and Methods

### Animals

The 23 primates (*Macaca mulatta*) used in this study were born and reared at the University of Pittsburgh (*N* = 19 female, *N* = 4 male). All animals were housed in group-rearing pen environments composed of 4–5 primates ranging in age from adolescence to adulthood. Animals were divided into cohorts, exposed to maternal separation, and behaviorally assessed. All experiments were approved by the University of Pittsburgh Animal Care and Use committee and were in accordance with guidelines issued by the National Institute of Health. Following sacrifice, brains were transferred to the University of Rochester for assessments. Tissue from some of these animals was previously used for amygdala gene expression and neuronal cell counting (Sabatini et al., 2007; de Campo et al., 2017; McHale-Matthews et al., 2023).

### Maternal separation paradigm

**Figure 1.**
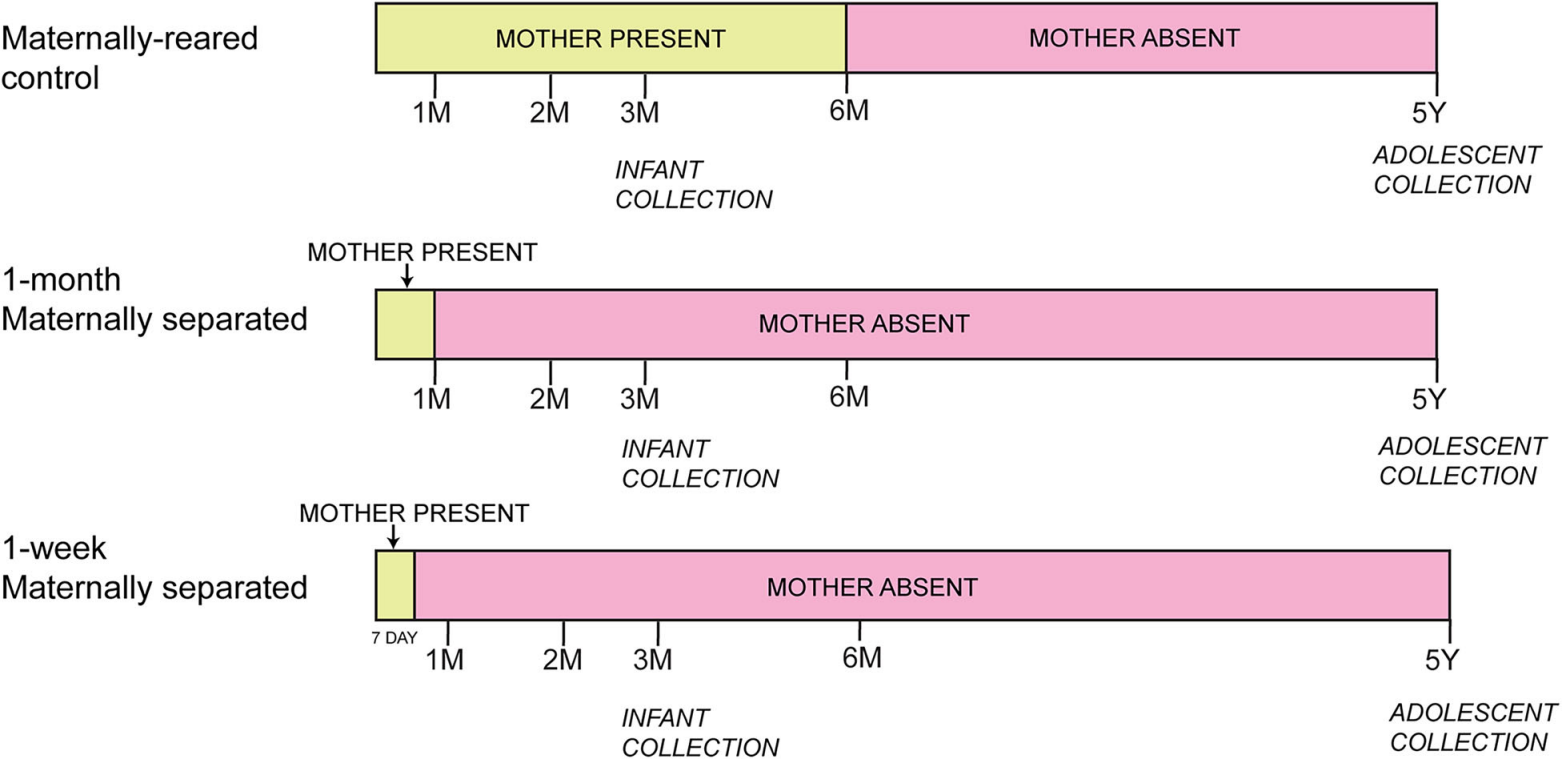
General design of the maternal separation paradigm in infants and adolescent animals. Yellow depicts mother present; pink depicts mother absent from group pen.

The maternal separation protocol has been extensively described in previous publications and is described here in brief. In this model, there were no effects on weight gain or food intake. Two age cohorts, an infant (3 months at sacrifice) and an adolescent (4–5 years at sacrifice) were exposed to similar protocols beginning at birth (Fig. 1). In the infant cohort (killed at 3 months), all animals were female (*n* = 12). In the adolescent cohort (killed at 4–5 years), there were three males and eight females (*n* = 11).

All animals shared a single cage with their mother during the first week of life (per typical animal husbandry protocols) and were then randomly assigned to one of the following experimental groups: “1-week separated (1-WS),” “1-month separated (1-MS),” or “maternally reared (MR).” The randomization to two separation groups was based on the behavioral impact of the timing of maternal separation in monkeys (Sabatini et al., 2007; Cameron et al., 2017). Maternally reared animals were reared in the group pen with mother for 3 months (infant) or 6 months (adolescents), after which the mother was removed (Fig. 1). 1-WS animals in both infant and adolescent cohorts were in group pen with mother for 1 week. Similarly, 1-MS animals in both infant and adolescent groups were in the group pen with the mother for 1 month (infant and adolescent). After the mother was removed from the pen, separated animals remained in the pen with other animals. For full details of the animal husbandry and the modified maternal separation paradigm, see (Sabatini et al., 2007; de Campo and Fudge, 2012; Cameron et al., 2017; McHale-Matthews et al., 2023).

#### Group-rearing pen environment

At the beginning of the study, only one experimental infant and its mother were present in each group-rearing pen, with three nonexperimental monkeys of varying ages (juvenile to adolescent in age). When the mother was present, her status was therefore the most dominant member of the group, to insure there was no added stress on the mother. Maternally reared (MR) and 1 month separated (1-MS) animals were introduced to a group-rearing pen environment with their mother present from the beginning of Week 2. The 1-WS group had their mother removed but was left in the single cage and taught to bottle feed for a 5–7 d period (*ad libitum* Similac with Iron baby formula; Abbott Laboratories). They were then placed in the pen at Week 2 of age and a hutch with a door small enough for the separated infant to transgress, but too small for other animals to enter, was placed in the pen. Bottles were made available to the infant in this cage. A soft, cotton-stuffed toy was placed in the cage to provide contact comfort. In contrast, 1-MS animals were integrated into a group-rearing pen environment with their mother present for Weeks 2–4. At the end of Week 4, the 1-MS animals were separated from their mothers and trained to bottle feed in a single cage adjacent to the pen for a 1 week period and then re-introduced to the same group-rearing environment for Weeks 5–12. We analyzed the two separation groups individually to investigate possible timing effects of maternal separation. For the infant cohort, the MR group had their mother present in the pen for the 12 weeks of the study. For the adolescent cohort, maternally reared (MR) animals continued with the mother in the group-rearing pen through 6 months of age, since this is the typical point at which macaque mothers leave their infant and form a consort with a male macaque for the next mating season (Cameron et al., 2017).

For the adolescent cohort, monkeys were reared until they were 4.43 ± 0.16 years of age in the stable group pens which they were placed in at 2 weeks of age. They were then regrouped into new social groups and housed in mixed sex colonies. The new social groups consisted of one 1-WS, one 1-MS, and one 6-MS to study their behavioral response to entering a new social group. Adolescent monkeys were killed 8.95 ± 0.31 months after regrouping when they were 5.14 ± 0.17 years of age.

### Tissue collection and processing

Brain tissue from the infant cohort was harvested at Week 12. Brain tissue from adolescent animals was harvested at 4–5 years. All animals were first anesthetized with ketamine HCl (10 mg/kg, i.m.) and then deeply anesthetized with sodium pentobarbital (30 mg/kg, i.v.). A transcardial perfusion surgery protocol was followed, using the following: 1 L of ice-cold 0.9% NaCl solution containing 2% sodium nitrite and 5,000 IU heparin. The brain was removed, hemisected on the midsagittal plane. The left hemisphere used in this study was cut in coronal blocks and immersion fixed (Sabatini et al., 2007; de Campo et al., 2017). Blocks were sectioned coronally on a freezing sliding microtome at 40 μm thickness and the rostral to caudal extent of the amygdala was saved in serial compartments.

### Histology for fixed tissue

#### Immunocytochemistry

One of twelve sections from adjacent compartments were chosen to ensure rostral to caudal sampling of the entire amygdala, yielding ∼7–9 sections per case. Preliminary data in the PL (immature neuron) and basal nucleus (mature neurons) showed differing baseline microglia morphologies for these two regions (data not shown). We therefore focus first on microglia in the PL, which is unique for its immature neurons. Protocols for doublecortin (DCX) and ionized calcium binding adaptor 1 (IBA1) immunolabeling were established in control animals that were not part of this study (Fudge et al., 2012). DCX protein identifies immature neurons of the PL, distinguishing them from overlying basal nucleus (de Campo et al., 2017; Page et al., 2022; McHale-Matthews et al., 2023). IBA1 is a cytoplasmic protein found in macrophage-derived cells such as microglia (Ito et al., 1998). Cases were then immunolabeled in batches in a blinded counterbalanced fashion as follows.

Tissue was rinsed in PB with 0.3% Triton X-100 (PB-TX) overnight. The next day, the sections were first treated with an endogenous peroxidase inhibitor for 5 min and then rinsed in PB-TX for a total of six rounds, 15 min each round with the replacement of fresh PB-TX. After the rinses, sections were preincubated for 30 min in 10% normal donkey serum blocking solution with PB-TX (NGS-PB-TX). Sections were then incubated in primary antisera to DCX (1:15,000, Abcam, rabbit, AB18723) or IBA1 (1:5,000, Fujifilm, rabbit, 019-19741) at 4°C on a rocker for four nights. Sections were again rinsed, blocked with 10% NGS-PB-TX, and incubated for 40 min in anti-rabbit biotinylated secondary antibody. After secondary antibody incubation, and sequential rinses over 1.5 h, sections were incubated in an avidin-biotin complex for 1 h (Vectastain ABC Elite; Vector Laboratories) and then visualized with 3,3′-diaminobenzidine (DAB).

All immunolabeled sections were rinsed overnight to reduce background staining and then mounted onto gelatin-treated slides from 0.1 M PB solution and left to air-dry for 2–4 weeks. Once dry, DCX-labeled sections were additionally dehydrated, rehydrated, and then counterstained lightly with cresyl violet (Chroma-Gesellschaft). All sections were coverslipped with DPX Mounting Medium (Electron Microscopy Sciences).

### Analysis

#### Location of regions of interest

**Figure 2.**
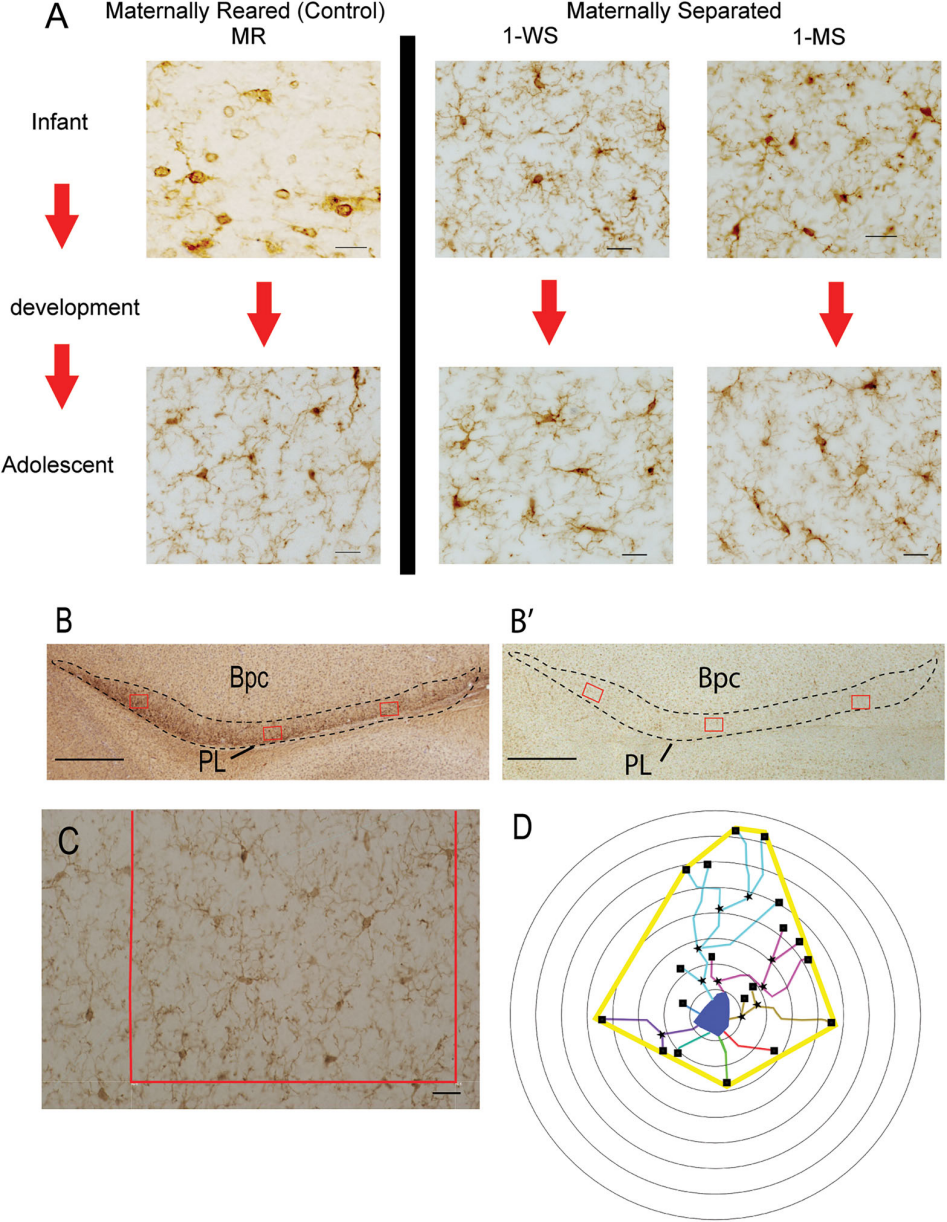
***A***, Overview of the experimental design. Photomicrographs of IBA1 + microglia in the PL across all experimental groups (scale bar, 25 μm). ***B***, Low power image of dense DCX-IR used to define the PL (contour) and 3 regions of interest (ROI). Scale bar, 500 μm. ***B’***, Adjacent section immunoreacted for IBA1, aligned using fiducial landmarks such as blood vessels, with the PL contour/ROIs superimposed. ***C***, High power image of ROI for collection of density, spacing index, and soma size data. Scale bar, 25 µm ***D***, Schematic of representative Sholl measurements. Blue delineates the cell soma, and individual processes originating at the cell soma are shown in assorted colors, black stars mark nodes and squares denote endings. The thick yellow line shows the convex hull measure.

The study was designed to compare microglia characteristics in infancy and adolescence, and the effect of maternal separation at either 1 week or 1 month of life on both infant and adolescent PL (Fig. 2*A*). All slides from each case were coded to ensure that investigators were blind to age and maternal separation condition. For each case, we assessed 4–5 evenly spaced IBA1-labeled slides, each aligned with adjacent sections immunolabeled for DCX-IR to localize the PL and matched for rostral to caudal level across animals. The PL was identified in DCX/Nissl-labeled sections using bright-field microscopy with a 2× objective (Fig. 2*B*). Intensely labeled DCX-positive immature neurons are seen in the laminar PL structure ventral to the basal nucleus, and a contour was drawn around this region using mapping software (Olympus AX70 microscope interfaced with Stereoinvestigator via a video CCD, MicroBrightField). Adjacent IBA1-labeled sections were captured at the same magnification, and contours were aligned onto matching landmarks such as blood vessels and fiber tracts to localize the PL (Fig. 2*B’*). Within the overlaid contour, the IBA1-labeled cells in PL in three regions of interest (ROIs) were photographed using a 40× objective (Fig. 2*C*). Images were initially collected in medial, central, and lateral ROIs for each section. However, in preliminary analyses (data not shown), we determined that there were no statistical differences in microglial density, spacing, or soma size across these regions in any animal. Therefore, data from these locations was pooled, averaged per animal, and then for the group, for PL microglia density counts, soma size, and spacing index values.

#### Microglial morphology data collection

##### Microglial density

During normal development, microglia density increases in many brain regions between infancy and adolescence (Menassa et al., 2022) and can also be altered by early life stress (Reemst et al., 2022). Therefore, we calculated the area of the PL by using the freehand tool in Fiji/ImageJ to retrace the previously outlined contour of the PL. All microglia within each ROI were selected using the multipoint tool, which also automatically records the *x*- and *y*-position of each selected microglia. Microglia density was calculated as the average total number of microglia per ROI area for each animal and averaged by group.

##### Spacing index

The spacing of the microglia was calculated to determine the nearest neighbor distance between individual microglia, corrected for density. During normal development microglia are typically evenly distributed, and in general, spacing does not significantly differ depending on age (Savage et al., 2019). The “spacing index” (or “clustering index”) is calculated as follows: (average nearest neighbor distance)^2^ × microglial density. The spatial coordinates captured (above) were entered into a customized MATLAB code (MathWorks) where the distance between each microglia and its nearest neighbor was determined and averaged for all microglia per ROI for each animal and averaged by group.

##### Soma area

The normal developmental transition from infancy to adolescence is often marked by decreased microglial soma size (Savage et al., 2019). However, in pathological conditions, this transition is broadly impacted (Reemst et al., 2022). Therefore, we examined a total of 80–100 microglia per animal, by hand-tracing from 40× images using the ImageJ freeform tool to outline the cell body. Using the analyze, “calculate area” function, the program then automatically calculated the area of the hand-drawn soma in each ROI and expressed it as square micrometer. Average soma size was averaged for each animal and averaged by group.

##### Morphological analyses

To determine measures of microglia complexity at different ages, and under different conditions, Sholl and related analyses were conducted on 20 randomly selected skeletonized microglia in the central PL of each animal. Using the overlaid contour on IBA1 sections, a sampling grid was used to help select a central ROI on each section (4–5 sections through the rostrocaudal PL/animal). A 40× image of the microglia within the grid box was then captured (Fig.2*C*), and the microglia contained inside the grid box were assigned a number. A random number generator (www.Calculator.net) was used to indiscriminately select five microglia in the 40× image for tracing. A total of 20 randomly selected IBA1-positive microglia per animal were thus drawn using the two-dimensional (2D) neuron reconstruction module (Neurolucida 360, MicroBrightField Bioscience). We marked soma, process length (total dendrite length), branch points, and endings for each cell. A 2D convex hull for each cell was created from a line connecting endings (Fig. 2*D*).

Sholl analysis parameters (Neurolucida Explorer, MicroBrightField Bioscience) were set to place concentric circles beginning at the center of the soma, in 5 μm intervals (Fig. 2*D*). The number of intersections at each interval from the soma was used to generate a Sholl profile curve, from which several indices of microglial morphology can be derived by the following: (1) convex hull area, (2) number of branching nodes, (3) number of endings, and (4) mean process length. The 2D convex hull area is the area enclosed around the planar shape of the tracing, and convex hull perimeter is the distance around the most distal points (endings) that form the convex hull. The total number of nodes and total number of endings in the shell were also calculated. The total length in micrometer of all processes passing through a shell was calculated as the mean process length. All these data were exported into an Excel file and averages were calculated first for each animal and then for the group.

### Statistics

Statistical tests were run with Prism 10 statistical analysis software (GraphPad). Maternally reared animals versus 1 week and 1 month separated animals in infant and adolescent groups were statistically analyzed using an ordinary two-way ANOVA with Tukey's multiple-comparisons tests, to directly assess morphologic changes in each group between maternally reared and separated groups. Pairwise comparisons and interactions between age and condition were considered significant if the *p* value associated with them was <0.05.

## Results

### Maternal separation alters microglial population characteristics in infants and adolescents

**Figure 3.**
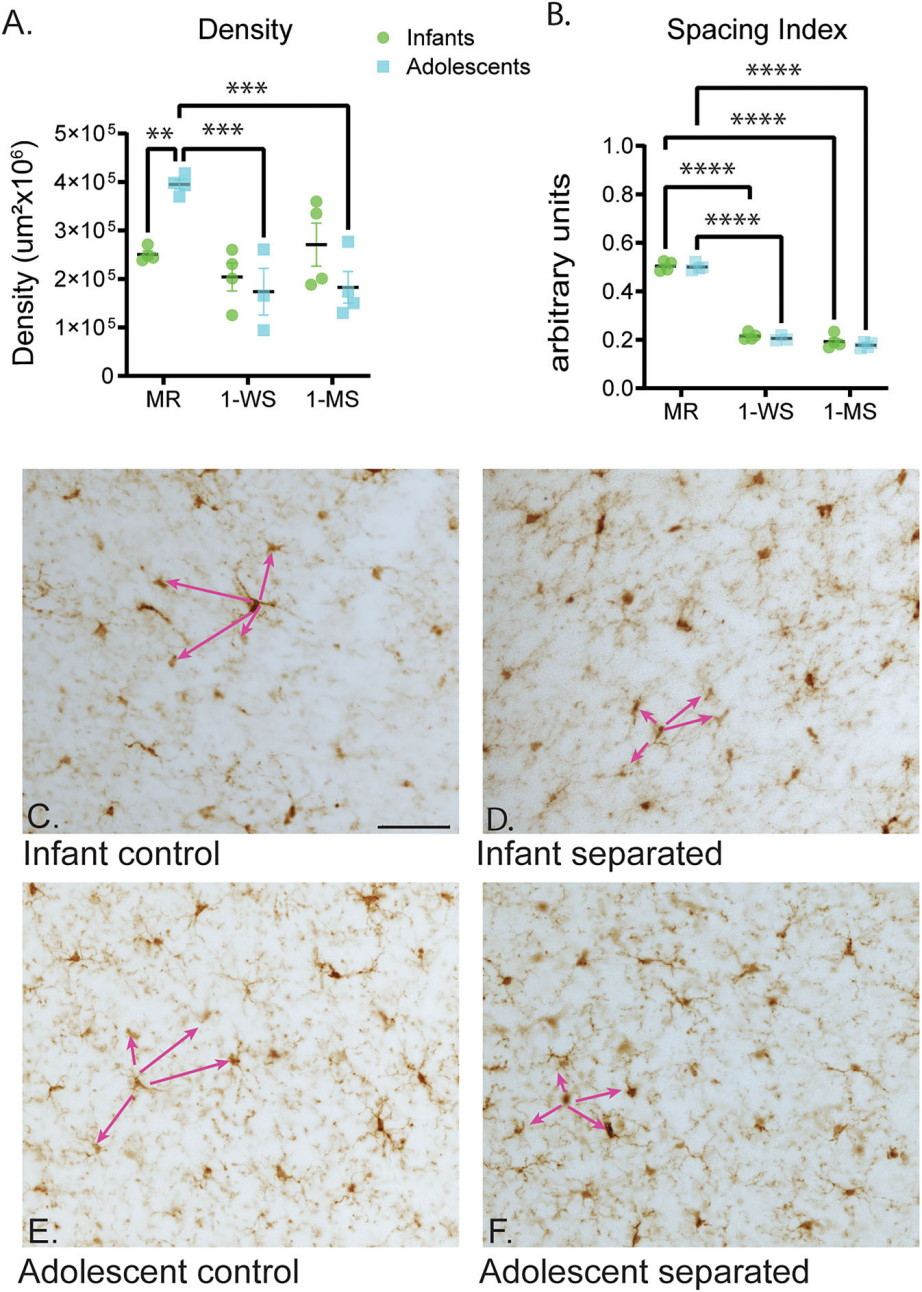
Maternal separation alters microglial population characteristics in infants and adolescents. The infants (green) and adolescents (blue) in all conditions are compared using the following measures: (***A***) density and (***B***) spacing index. ***C–E***, Representative images of microglia populations in infant and adolescent groups to show the concept of the “spacing index” (the distance of all microglia in the sample to the “nearest neighbors” is averaged and normalized for density). Lower values in ***B*** indicate shorter average distances or more clustering. For simplicity, pink arrows show just one example in each photomicrograph. Scale bar, 100 μm. *p < 0.05; **p < 0.01; **p < 0.001; ****p < 0.0001.

To provide an overview of microglia population characteristics, we first characterized how the global distribution of PL microglia differs between maternally reared (MR) infant and adolescent macaques (Fig. 3). The density of PL microglia was significantly increased in the MR adolescents (mean = 394,923 ± 19,771/µm^2^ × 10^6^) compared with MR infants (mean = 250,463 ± 14,324/µm^2^ × 10^6^; *p* = 0.0036; Fig. 3*A*).

We then examined changes in microglia from MR animals with those from the 1-WS (animals separated beginning at 1 week of life) and 1-MS (animals separated beginning at 1 month of life) animals for each age cohort. Overall, there were no differences in density between the MR infants (264,265 ± 14,799/µm^2^ × 10^6^) and either group of separated infants [the 1-WS infants (204,265 ± 57,934/µm^2^ × 10^6^; *p* = 0.5398) or 1-MS infants (270,904 ± 88,981/µm^2^ × 10^6^; *p* = 0.8829); Fig. 3*A*]. However, in adolescents, the overall density of PL microglia was markedly decreased in the 1-WS (mean = 17,3621 ± 83675/µm^2^ × 10^6^; *p* = 0.0005) and 1-MS (mean 18,2674 ± 65,292/µm^2^ × 10^6^; *p* = 0.0003), when compared with the MR adolescents (mean 394,923 ± 19,771 µm^2^ × 10^6^; Fig. 3*A*).

The spacing index value, which measures the clustering of neighboring microglia, was similar between MR infants (0.5039 ± 0.02052 AU) and adolescents (0.5003 ± 0.01494 AU; *p* = 0.7867; Fig. 3*B*). This indicated that, despite the increase in microglia density with development, when corrected for density, microglia are evenly distributed in MR infant and adolescent PL.

In infants, the spacing index was markedly decreased in both the 1-WS (0.2146 ± 0.01376 AU; *p* < 0.0001) and 1-MS (0.1931 ± 0.02913 AU; *p* < 0.0001) groups in comparison with the MR group (0.5039 ± 0.02052 AU; Fig. 3*B*). This suggested that the microglia have a more clustered distribution, despite similar densities between maternally reared and maternally separated infants. This substantial decrease in spacing index value was also observed in both the 1-WS (0.2058 ± 0.01126 AU; *p* < 0.0001) and 1-MS (0.1776 ± 0.01240 AU; *p* < 0.0001) adolescent groups compared with the MR adolescents (0.5003 ± 0.01494 AU; Fig.3*B*). This indicates that increased clustering was associated with maternal separation in both age groups (Fig. 3*C–E*).

### Maternal separation alters microglial morphology in infants and adolescents

**Figure 4.**
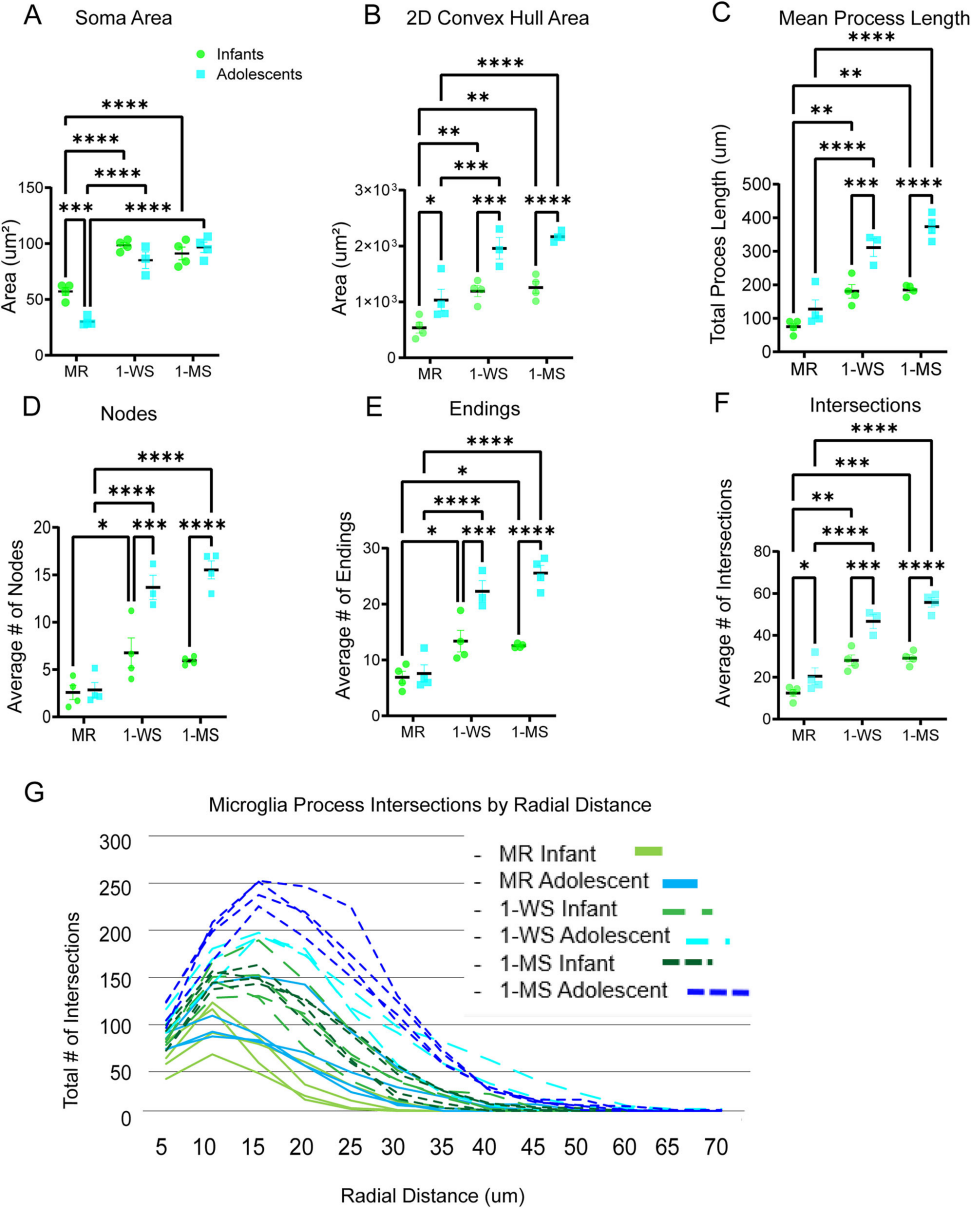
Maternal separation alters microglial morphology in infants and adolescents. The infants (green) and adolescents (blue) in all conditions are compared using the following measures: (***A***) soma area. ***B***, 2D convex hull area. ***C***, Mean process length. ***D***, Average number of nodes. ***E***, Average number of endings. ***F***, Average number of intersections. ***G***, Microglia process intersections by radial distance (infants, green; adolescent, blue; MR, solid line; 1-WS, fragmented lighter colored line; 1-MS, fragmented darker colored line). *p < 0.05; **p < 0.01; **p < 0.001; ****p < 0.0001.

We then focused on the morphological features of the microglia. The soma area of microglia was significantly greater in MR infants (mean = 57.14 ± 7.088 µm^2^) compared with MR adolescents (mean = 30.15 ± 3.944 µm^2^; *p* = 0.0003; Fig. 4*A*). After maternal separation, soma area was further increased in both the 1-WS (mean 98.40 ± 4.975 µm^2^; *p* < 0.0001) and 1-MS (mean 91.12 ± 11.30 µm^2^; *p* < 0.0001) infant groups compared with the MR infant group (mean 57.14 ± 7.088 µm^2^; Fig. 4*A*). Surprisingly, this pattern was also seen in adolescents, with soma size also enlarged in both the 1-WS (mean = 85.07 ± 12.92 µm^2^; *p* < 0.0001) and 1-MS (mean = 96.59 ± 9.280 µm^2^; *p* < 0.0001) compared with the MR adolescent groups (mean = 30.15 ± 3.944 µm^2^; Fig. 4*A*).

In the MR groups, we found that the average convex hull area, which measures the entire area occupied by the microglia, is significantly greater in the MR adolescents (1,032 ± 385.4 µm^2^) compared with the MR infants [536.7 ± 190.3 µm^2^; *p* = 0.0119; Fig. 4*B*; similar to the convex hull perimeter (infants = mean 92.38 ± 14.23, adolescents = mean 130.6 ± 19.46; *p* = 0.0009); data not shown]. In infants, maternal separation resulted in increased convex hull area in both 1-WS (mean = 1,191 ± 196.7 µm^2^; *p* *=* 0.0046) and 1-MS (mean = 1,257 ± 210.7 µm^2^; *p* = 0.0.0021) groups compared with MR infants (mean = 536.7 ± 190.3 µm^2^; Fig. 4*B*). As expected, convex hull perimeter was similarly increased in the 1-WS (mean 136.1 ± 10.9 μm; *p* *=* 0.0008) and 1-MS (mean = 138.3 ± 13.95 µm; *p* = 0.0005) groups in comparison with the MR infant group (92.38 ± 14.23 μm; data not shown). A larger 2D convex hull area was also seen in maternally separated adolescents [1-WS (mean = 1,958 ± 335.5 µm^2^; *p* = 0.0004); 1-MS (mean = 2,166 ± 87.39 µm^2^; *p* < 0.0001)] in comparison with that in MR adolescent group (mean = 1,032 ± 385.4 µm^2^). The 2D convex hull perimeter measurements (data not shown) were also greater in the 1-WS (mean = 172.5 ± 13.90 µm; *p* = 0.0022) and 1-MS (mean = 178.6 ± 3.326 µm; *p* = 0.0003) groups relative to the MR adolescent group (mean 130.6 ± 19.46 µm).

Next, we examined the mean total process length, which sums the length of all the processes passing through the convex hull. In the MR animals, there was a trend for mean total process length to be greater in the adolescents (127.5 ± 55.39) in comparison with the infants (75.08 ± 20.39; *p* = 0.0677), which did not reach significance (Fig.4*C*). However, the mean total process length increased in both groups with maternal separation, consistent with the observed larger convex hull area and perimeter. In infants, the mean process length was significantly increased in both 1-WS (mean = 180.7 ± 40.55 μm; *p* = 0.0029) and 1-MS (mean = 184.6 ± 16.14 μm; *p* = 0.0022) compared with the MR infants (mean = 75.08 ± 20.39 μm). This was also seen in adolescents, where mean total process length was also significantly higher in both separation groups [the 1-WS (mean = 311.1 ± 46.09 µm; *p* *<* 0.0001);1-MS (mean = 373.6 ± 37.06 µm; *p* < 0.0001)] compared with MR adolescents (mean = 127.5 ± 55.39 µm; Fig. 4*C*).

To further characterize the complexity of the microglia, we determined the mean number of nodes, which measures the mean number of bifurcations of an individual microglial process. In the MR animals, the mean number of nodes in the infants (mean = 2.575 ± 1.487) and adolescents were similar (mean = 2.838 ± 1.554; *p* = 0.8517; Fig. 4*D*). However, after maternal separation the mean number of nodes between the MR (2.575 ± 1.487) and the 1-WS (6.750 ± 3.159; *p* = 0.0200) was increased. However, mean number of modes was not significantly elevated in the 1-MS (5.925 ± 0.4291; *p* = 0.0658) infants. The mean number of nodes also dramatically increased between the MR (2.838 ± 1.554) and both the 1-WS (13.67 ± 2.226; *p* < 0.0001) and 1-MS (15.53 ± 1.891; *p* < 0.0001) adolescent animals.

The mean number of endings followed patterns observed for nodes described above. While the mean endings were similar in the MR infant (mean = 6.888 ± 2.171) and adolescent (mean = 7.575 ± 3.084; *p* = 0.7320) groups (Fig. 4*E*), the mean number of endings greatly increased with maternal separation in both infants and adolescents. In infants, both the 1-WS (13.36 ± 3.873; *p* = 0.0117) and 1-MS (12.54 ± 0.3816; *p* = 0.0277) had significantly more endings compared with MR control (6.888 ± 2.171). This pattern of increased endings was also observed in the adolescent groups where the mean number of endings more than tripled between the MR (7.575 ± 3.084) and both the 1-WS (22.28 ± 3.299; *p* *<* 0.0001) and 1-MS (5.54 ± 2.754; *p* < 0.0001) adolescent animals.

Finally, the mean number of intersections, which indexes crossings of the Sholl and is a measure of branching, was increased between the MR infants (12.46 ± 3.347) and adolescents (20.46 ± 7.846; *p* = 0.0439). Maternal separation in both infants and adolescents increased total mean intersections. In infants, the mean number of intersections in the 1-WS (28.05 ± 5.203; *p* = 0.0015) and 1-MS (29.11 ± 3.271; *p* = 0.0008) groups were more than double the mean number of intersections in the infant MR group (12.46 ± 3.347; Fig.4*F*). Similarly, the 1-WS (mean = 55.39 ± 5.811; *p* < 0.0001) and 1-MS (mean = 55.68 ± 4.445; *p* < 0.0001) adolescent groups have more than double the mean intersections found in the MR adolescents (mean = 20.46 ± 7.864).

To examine the arbor complexity of microglia in more detail, we mapped the frequency of intersections between the processes and spheres at 5 µm incremental radii from the soma for each animal in each group (Fig.4*G*, green, infants; blue, adolescents). This shows the point of peak intersections for each animal, and the frequency of tapering of intersections at greater distances from the soma. Maximal intersections in infant (solid green line) and adolescent (solid blue line) maternally reared animals were greatest at ∼10 μm from the soma. Microglia arbors ended ∼20–25 µm from the soma in MR infant PL, whereas in the MR adolescent PL, intersections were further extended out to ∼35–40 µm radial distance, supporting an overall greater distal elongation of processes in the MR adolescent PL, also seen in convex hull data (Fig. 4*B*). While the microglial arbors in the MR infant PL (green) extended to 25–30 µm, in both the 1-WS (light green fragmented line) and 1-MS (dark green fragmented line) infants, arbors extended further, maximally between 35–45 µm away from the soma. Similarly, in adolescents (blue), the most distal intersections of the microglia processes were less in the MR (∼40 µm), compared with 1-WS (∼50 µm) and 1-MS (∼55 µm) adolescent animals. As can be seen in both age groups, maternal separation (all fragmented curves) resulted in increased microglial process intersections at all distances compared with MR but was highest in the adolescent animals, evidenced by the tallest peaks occurring in both 1-WS and 1-MS adolescent groups (blue fragmented lines). In general, adolescent animals separated at 1 week and 1 month have the largest arbors and increased intersections demonstrating increased microglial complexity (Fig. 4*G*).

Lastly, there were no statistical differences in any measures between the 1-WS and 1-MS animals in either infant or adolescent cohort: infants, density (*p* = 0.2913), spacing index (*p* = 0.2534), soma size, (*p* = 0.4682), 2D convex hull area (*p* = 0.8391), mean process length (*p* = 0.9894), intersections (*p* = 0.9633), nodes (*p* = 0.8039) and endings (*p* = 0.9088) and adolescents, density (*p* = 0.9791), spacing index (*p* = 0.1423), soma size (*p* = 0.2124), 2D convex hull area (*p* = 0.6617), mean process length (*p* = 0.5130), intersections (*p* = 0.3821), nodes (*p* = 0.2539), and endings (*p* = 0.3043).

### Maternal separation disrupts the normal developmental trajectory of microglia

**Figure 5.**
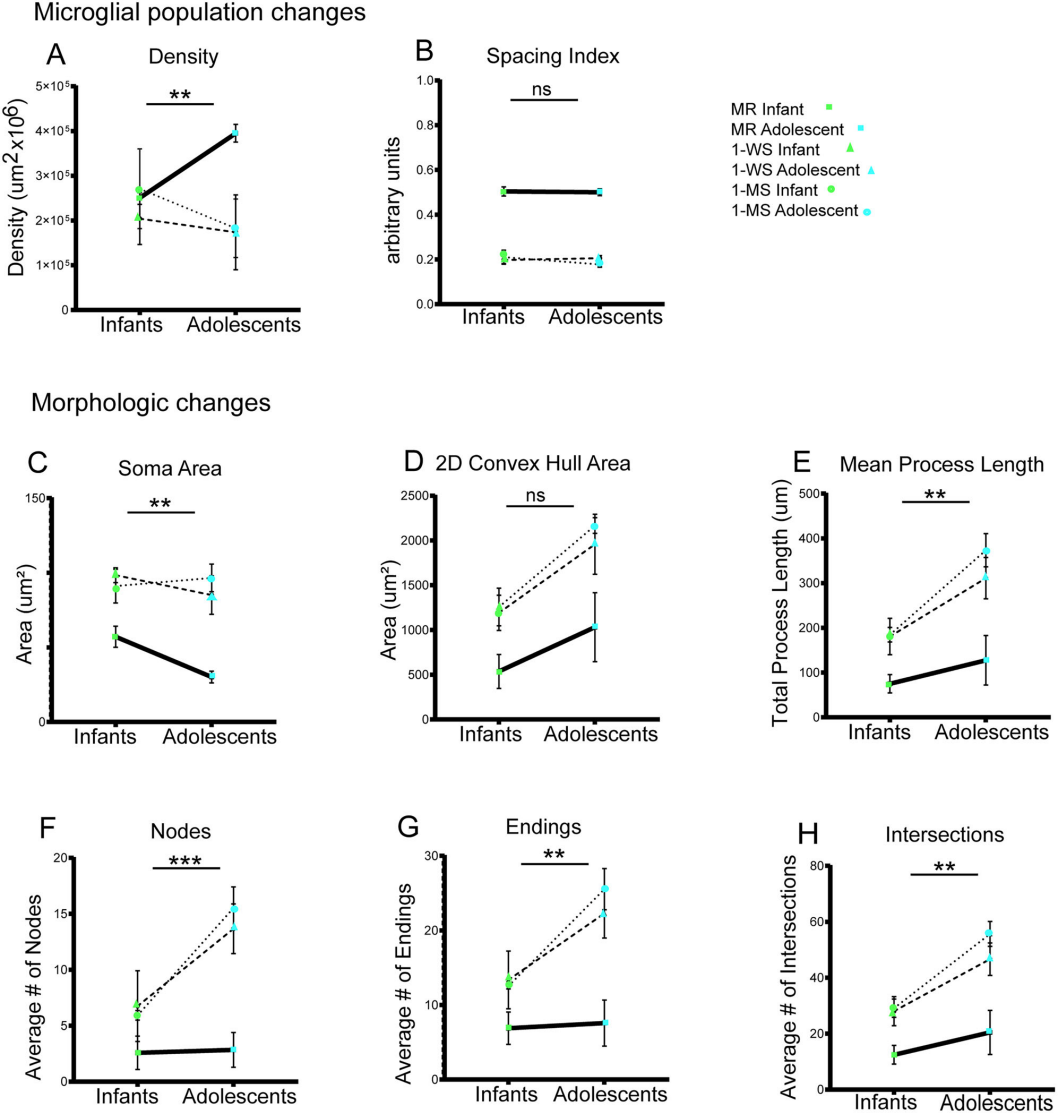
Interactions between maternal separation (experimental condition) and age. Graphs show the comparisons among all experimental conditions between infancy and adolescence (infants, green; adolescents, blue) in microglial population and morphology. The experimental conditions are distinguished as follows: MR animals (shown with squares and solid line), 1-WS animals (shown with triangles and heavy dashed line), and1-MS animals (shown with circles and dotted line). Asterisks indicate significant interactions between the factors of age and experimental condition *p < 0.05; **p < 0.01; **p < 0.001; ****p < 0.0001. See text for significance of individual factors.

To understand whether maternal separation, age, or the combination of both factors impacted the transition from infancy to adolescence, we examined interactions between the two factors: maternal separation and age (infant, adolescent; Fig. 5*A–H*).

#### Microglial population changes

The normal increase in microglial density with adolescence was blunted by maternal separation (age × experimental condition; *F*_(2,17)_ = 7.940; *p* = 0.0037). Maternal separation (*F*_(2,17)_ = 9.824; *p* = 0.0015) and not age (*F*_(1,17)_ = 0.1126; *p* = 0.7413) was the driving factor in this significant interaction (Fig. 5*A*). The microglial spacing index remained similar between infancy and adolescence in all groups (*F*_(2,17)_ = 0.1957; *p* = 0.8241) but was uniformly decreased in both infant and adolescent maternally separated groups (experimental condition; *F*_(2,17)_ = 17.79; *p* < 0.0001). There were no age-dependent changes to the spacing index value (*F*_(1,17)_ = 1.488; *p* = 0.2423), indicating that maternal separation alone is associated with increased clustering that persists into adolescence (Fig. 5*B*).

#### Morphologic changes

Both age and maternal separation influenced the morphological complexity of microglia. The normal reduction in soma size between infancy and adolescence was lost in 1-WS and 1-MS animals, accounted for by overall increases in soma size in all separated animals compared with their MR controls. Both age (*F*_(1,17)_ = 10.46; *p* = 0.0049) and maternal separation (*F*_(2,17)_ = 86.00; *p* < 0.0001) interacted to impact the soma size (age × experimental condition; *F*_(2,17)_ = 7.234; *p* = 0.0053; Fig. 5*C*).

The 2D convex hull, which normally increases with age, showed a similar shift and increased further at both ages in both separated groups (Fig. 5*D*). Age (*F*_(1,17)_ = 48.13; *p* < 0.0001) and maternal separation (*F*_(2,17)_ = 31.97; *p* < 0.0001) factors independently contributed to the changes observed in hull area (age × experimental condition interaction; *F*_(2,17)_ = 1.429; *p* = 0.2670). Age (*F*_(1,17)_ = 45.70; *p* < 0.0001) and maternal separation (*F*_(2,17)_ = 29.09; *p* < 0.0001) also independently impacted the perimeter (*F*_(2, 17)_ = 0.03951; *p* = 0.9613; data not shown).

For the mean process length (Fig. 5*E*), nodes (Fig. 5*F*), and endings (Fig.5*G*), maternal separation interacted with development (age) to result in significant increases in these three measures between infant and adolescent groups [age × experimental condition: mean process length (*F*_(2,17)_ = 6.504; *p* = 0.0080), nodes (*F*_(2,17)_ = 12.02; *p* = 0.0006), endings (*F*_(2,17)_ = 10.05; *p* = 0.0013)]. This indicates that both maternal separation and age increase these measures of microglial complexity. Maternal separation significantly contributed to the changes in the mean process length (*F*_(2,17)_ = 48.96; *p* < 0.0001), nodes (*F*_(2,17)_ = 41.29; *p* < 0.0001), and endings (*F*_(2,17)_ = 42.49; *p* < 0.0001). The increases in these measures were also affected by age, with greater values in adolescents compared with infants [mean process length (*F*_(1,17)_ = 60.51; *p* < 0.0001), nodes (*F*_(1,17)_ = 46.51; *p *= <0.0001), endings (*F*_(1,17)_ = 41.38; *p* < 0.0001)].

The enhancement in total intersections beyond simple age effects was also driven by an interaction between age and experimental condition factors (*F*_(2,17)_ = 6.416; *p* = 0.0084; Fig. 5*H*). Both maternal separation (*F*_(2,17)_ = 55.36; *p* < 0.0001) and age (*F*_(1,17)_ = 66.08; *p* < 0.0001) were significant.

**Figure 6.**
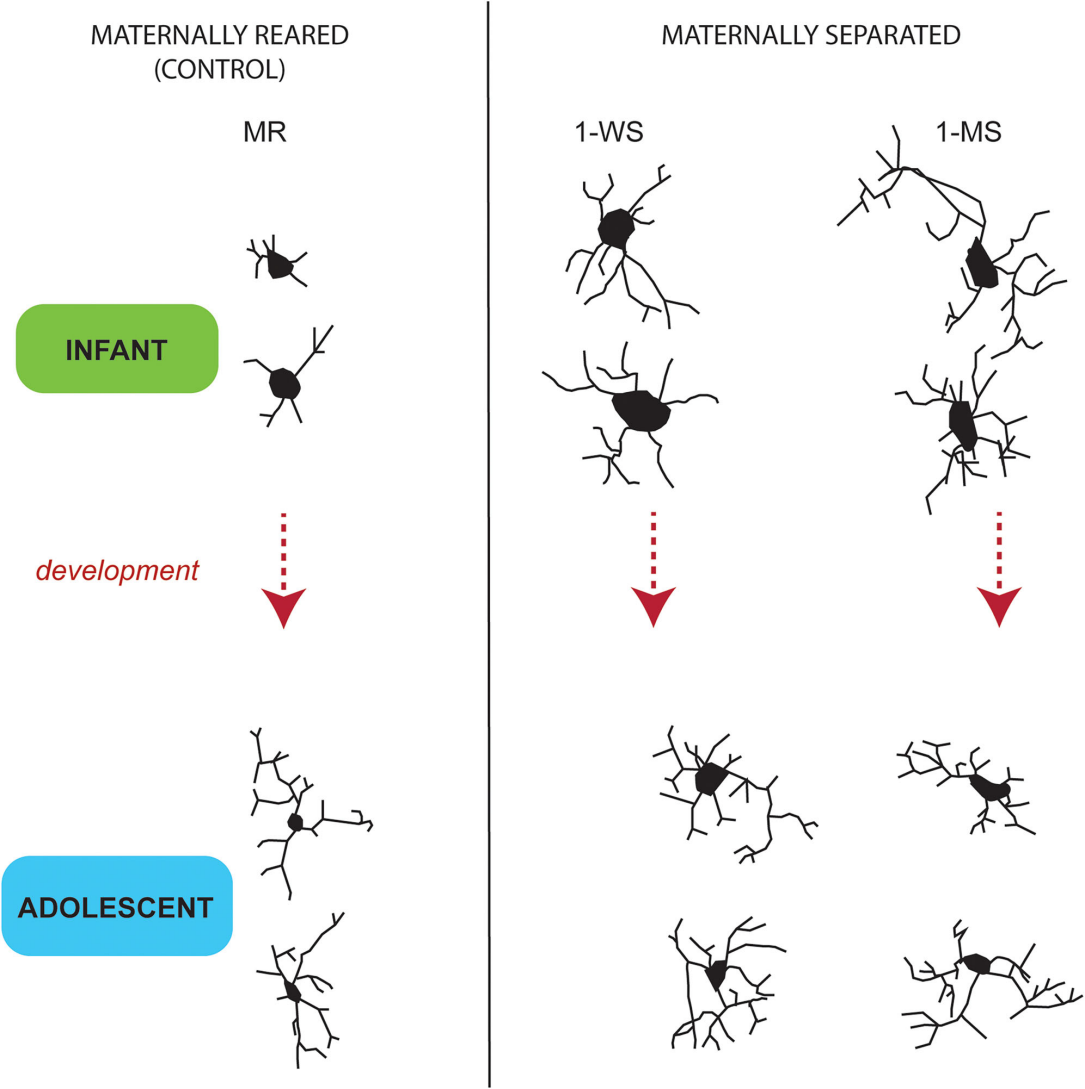
Maternal separation alters the normal developmental trajectory of PL microglia. Camera lucida drawings of typical microglia in maternally reared infants and adolescents compared with 1-WS and 1-MS in each cohort. Normal microglia development is associated with decreased soma size and increased microglial arborizations. Maternal separation results in an enlarged soma and increased microglial arborization and complexity in both infant and adolescent PL, suggesting that hyper-ramification persists into adolescence. Drawn with 40× objective.

Together, changes in convex hull, mean process length, nodes, endings, and intersections all suggest that the natural increase in microglia complexity with age is aberrantly heightened by maternal separation (Fig.6). For the majority of these measures, there was a significant interaction between age factors and separation condition.

## Discussion

The PL is a newly recognized source of cellular plasticity in the amygdala. We hypothesized that the morphology of PL microglia embedded among immature neurons would track shifts in neuronal growth during development. We also hypothesized that maternal separation is a potent environmental event that could dynamically alter this trajectory, with implications for neural growth and circuit formation.

### Normal PL development

Immature glutamatergic neurons of the PL are likely to depend on microglia for synapse formation, like developing neurons in other brain regions (Streit, 1993; Paolicelli et al., 2011). We investigated multiple measures of microglia morphology in the PL of both infant and adolescent animals to gain insight into functional state. In maternally reared animals, the PL microglia population density increased between infant and adolescent animals and maintained a homogenous distribution. At the same time, microglia soma size decreased, while their overall arbor area increased between infancy and adolescence. These findings are consistent with the typical developmental transition of microglia from an “immature” to “mature” phenotype (Savage et al., 2019; Dos Santos et al., 2020; Vidal-Itriago et al., 2022).

Prior literature has characterized the predominant microglial morphology during infancy having enlarged soma size and minimal processes (“amoeboid”; Ivy and Killackey, 1978; Innocenti et al., 1983). In this phenotype, microglia scavenge and phagocytose whole cells and have a high migratory capacity [see review, Vidal-Itriago et al. (2022)]. By adolescence, the distinctly smaller soma size, and elongated processes, appear closer to a “ramified” phenotype (Vidal-Itriago et al., 2022). This transition is consistent with the morphological shifts we observed in the microglia of maternally reared animals in the PL. Between infancy and adolescence, there was a substantial decrease in microglial soma size, along with an increase arbor size. While microglia in the ramified phenotype do not typically engage in whole cell phagocytosis, they are thought to surveil their surrounding parenchyma and phagocytose synaptic elements, a process known as synaptic pruning (Tremblay et al., 2010). During brain development, microglia interface with activity-dependent changes in synapse formation to eliminate weakened synapses (Whitelaw et al., 2023). Since the PL is packed with immature glutamatergic neurons that gradually mature between infancy and adolescence (presumably to integrate into amygdala circuitry; McHale-Matthews et al., 2023), it is notable that microglia density increases, and morphology shifts to a more ramifying (pruning) phenotype, during this time.

### Maternal separation effects on PL

Maternal separation is a potent environmental disruption for young animals, including macaques and humans (Seay et al., 1962; Seay and Harlow, 1965; Coe et al., 1988; Fabricius et al., 2008; Koe et al., 2016; Cameron et al., 2017). The modified model used here is more ethologic than older models using isolation or peer-rearing. Indeed, it replicates human and monkey real-world separations in which infants receive support from their social group after the mother leaves the group (Cameron, 2001).

Surprisingly, maternal separation had a large effect not only on the PL microglia in infants (separated from the mothers for a few weeks), but also on PL microglia in adolescents who had been separated from the mother many years prior. The effects of maternal separation were much larger than the typical age-related morphological changes observed in microglia between infancy and adolescence (Fig. 6). Microglia in the maternally separated animals at both ages had relatively enlarged somas and increased number of processes and mean process length, characteristics consistent with a “hyper-ramified” phenotype (Hinwood et al., 2013; Smith et al., 2019). The maintenance of this striking phenotype over years in the separated adolescents suggests that the effects of early maternal separation are long-lasting. Thus, early environmental influences, such as stress, that stimulate synaptic activity may alter developing neural circuitry in concert with microglia activity (Catale et al., 2020; Block et al., 2022).

### A persistent hyper-ramified microglia phenotype

The “hyper-ramified” phenotype, distinguished by increased microglial soma area, process length, and complex branching patterns (increased nodes, intersections, and endings), is observed when animals are subjected to chronic stress (Hinwood et al., 2012; Hinwood et al., 2013; Smith et al., 2019). The hyper-ramified subtype has been conceptualized as an “intermediate phenotype” (Streit, 1996; Walker et al., 2014) that may arise before the emergence of the classic “hypertrophic” phenotype associated with classic inflammation (a “bushy” morphology with retracted, thickened dendritic processes and large soma; Streit, 1996). Consistent with this concept, recent work indicates that the effects of stress on microglial ramification can occur without accompanying evidence of inflammation (e.g., increases in interleukin-1beta, MHC-II, CD68, terminal deoxynucleotidyl transferase dUTP nick end labeling, and activated caspase-3; Smith et al., 2019). Interestingly, rodents bred to be “low responders” to novelty (novelty-averse) have more “hyper-ramified” microglia than “high responders,” suggesting that in the nonchallenged state, animals that are inherently stress sensitive have this phenotype (Maras et al., 2022). In humans, the “hyper-ramified phenotype” is among the diverse microglial phenotypes commonly found in the normal adult brain (as assayed in the anterior cingulate; Torres-Platas et al., 2014a). However, in the brains of people who suffered from depression and died by suicide, the prevalence of “hyper-ramified” microglia is significantly increased (Torres-Platas et al., 2014b).

### Microglia–neuron interactions following stress

Microglial shifts in the face of stress have recently become viewed as adaptive homeostatic responses, which correlate with the duration and severity of the stressor (Walker et al., 2014; Woodburn et al., 2021). These responses are termed “para-inflammation” since classic neuroinflammatory responses such as macrophage activation, pro-inflammatory cytokine production, leukocyte recruitment, and tissue damage may not be present (Woodburn et al., 2021). During para-inflammation, the immune system sustains low activation to support homeostatic function. A proposed mechanism for “para-inflammatory responses” is the increased activity in glutamatergic neurons during stress and the increase in glucocorticoids, which in turn triggers compensatory responses in microglia along with shifts in their morphological phenotype. Accordingly, the hyper-ramified phenotype is functionally associated with experience-dependent modifications in several brain regions in rodent models and can impact synaptic formation and maintenance (Hinwood et al., 2013; Wohleb et al., 2018; Smith et al., 2019; Bollinger et al., 2023). In this conceptual model, increased glutamate release induced by chronic stress facilitates microglial synaptic phagocytosis, which can be seen as a protective (adaptive) mechanism (Woodburn et al., 2021). Here, we hypothesize that a shift to “hyper-ramification” after maternal separation could reflect protective microglial responses to facilitate neuronal survival, in this densely populated region of immature glutamatergic neurons.

Supporting the idea compensatory neuronal-microglial interactions in responses to stress, we recently showed in this cohort of infant monkeys PL-specific decreases in neuronal gene transcripts associated with spinogenesis, axogenesis, and migration during maternal separation (de Campo et al., 2017). For example, mRNA for the activity-dependent gene TBR1 which is required for glutamatergic spine formation (Darbandi et al., 2018) was strongly downregulated in the maternal separation condition. As TBR1 is normally highly expressed in the PL, its decrease at the mRNA level in maternally separated infants may reflect synapse loss due to microglial hyper-ramification/phagocytosis and loss of excitatory transmission. Alternately, reduction of TBR1 may also be a cell autonomous protective mechanism. Therefore, more exploration of gene expression changes in both infant and adolescent PL is clearly needed to understand PL neuron-microglial interactions after maternal separation.

One of the most surprising findings of our study was that “hyper-ramified” microglia were a dominant phenotype in maternally separated adolescent animals, many years after their separation. Although the mechanism of this persistent phenotype is still unclear, the phenomenon is reminiscent of an exacerbated “hyper-ramified” phenotype in individuals with history of major depression (Torres-Platas et al., 2014a; Torres-Platas et al., 2014b).The idea that microglia “priming” by prior stress has long-lasting effects on microglia morphology and function has been demonstrated in rodent models and is important for understanding later-developing psychiatric and neurologic illnesses (Catale et al., 2020).

#### Limitations

There are a few inherent limitations to this study, most driven by the value and labor involved in studying the macaque species such as small sample sizes. Although we were able to determine strong changes in microglial morphology, a larger sample may have permitted detection of subtle differences between the 1-WS and 1-MS conditions. A total of 19 female and four male monkeys were utilized in this study. In future work, sex differences in the effects of maternal separation on PL microglia in male and female macaques should be explored. Regrouping of all adolescent animals in the last year of life may have increased stress, with unknown effects on microglial morphology. Nonetheless, clear shifts in microglia in the infant cohort by maternal separation were similar, and frequently enhanced, in the adolescent separation group, and there was insignificant effect on adolescent maternally reared animals. This suggests that while later “re-grouping” stress (as well as other intervening life experiences) may have affected microglia in adolescent PL, maternal separation was required for the “hyper-ramified” phenotypes observed.

Finally, conclusions on what is functionally occurring with the microglia cannot be solely based on their morphology. Although we have identified dynamic developmental morphologic differences between our experimental groups, future studies will be needed to investigate the phagocytic state of the microglia and the engulfment of putative synapses.

### Conclusion

In conclusion, our results show that maternal separation induced an altered developmental trajectory of the microglia in the macaque PL. These changes persist in adolescence along with ongoing behavioral abnormalities. Because environmental challenges and activity influences have been shown to interfere with the development of microglia including their phagocytic activity, microglial changes due to maternal separation may in turn also alter PL neuronal maturational processes thought to support emerging amygdala function in young animals.

## Synthesis

Reviewing Editor: Julie Bakker, University of Liege Decisions are customarily a result of the Reviewing Editor and the peer reviewers coming together and discussing their recommendations until a consensus is reached. When revisions are invited, a fact-based synthesis statement explaining their decision and outlining what is needed to prepare a revision will be listed below. The following reviewer(s) agreed to reveal their identity: Susana Torres-Platas. Note: If this manuscript was transferred from JNeurosci and a decision was made to accept the manuscript without peer review, a brief statement to this effect will instead be what is listed below.

The paper has been seen by 2 reviewers, please find their comments below. However, play particular attention to the statistics being used and their description, since it is rather confusing which tests were actually used. First it is mentioned that unpaired t-tests were used, but in order to do a t-test, the normality of the data needs to be assessed. There is no mention in the statistics section that a normality test was performed. Second, with the number of t-tests performed, a correction should have been done for multiple comparisons, which was not done apparently. Finally, additional ANOVAs were done so why were the t-tests performed if everything could have been solved with the ANOVAS?

Reviewer 1:

I congratulate the authors for this nice finding and making this important contribution to the field. I have some more comments:

I have a concern regarding the 2D reconstruction of microglial cells. Neurolucida is made to reconstruct in 3D given that these cells display they morphology in 3D. Doing only 2D reconstructions might not be as accurate. If the authors have done all this work to do this, why did they do only 2D reconstructions?

-Line 234- "Adjacent IBA1-labeled sections were captured at the same magnification, and contours were aligned onto matching landmarks". I have a concerns about the use of DCN in an adjacent slice to trace the ROI.

Were they the adjacent slices? How big is the structure? I am wondering how the morphology of the structure changes in the following slice? If each slice is 40 um thickness what are the chances that the structure is the same in the following slice?

Why didn't the authors do a double IHC instead? They could have used just fluorescence to delineate the structure in the same slice. Although this is not critical for this paper, I believe this might be critical if anatomical claims.

- I had an initial concern about the morphological analysis been done at 40x. Changes may not be accurate with that magnification. However, the authors were able to detect reliable changes between their groups, showing that 40x is more than enough to detect changes in microglial morphology.

Line -199 they mentioned they found deferring microglia morphology between the basal nucleus and PL. Although it is said "not shown", I think is important to show it. If it is not central to the message fo the paper it can go in supplemental.

Figure 3- Is way too busy, if comparisons are NS is implicit when you are just showing significant differences denoted by asterisks. The figure might be easier to follow iof all the lines of NS comparisons are removed. Also, it would be helpful to show what is the meaning of the colors in the figure, not only in the legend. This is true for the rest of the figures.

Figure 6 is not mentioned in the text.

Other minor observations:

Line 119- the sentence: "In view of the extensive literature confirming that early environment influences -represent a major risk..." it might be good to change influences for another wors. As is, it is hard to understand the sentence. Maybe change influence for adversity or factors.

Line 139- It would be helpful to explain the groups on Figure 1 "Maternally reared animals were reared in the group pen with mother for 3 months (infant) or 6 months (adolescents), after which the mother was removed" It is quite confusing, maybe add that the 3 month is because the animals were sacrificed (infant collection).

Reviewer 2:

This study led by Julie Fudge report significant differences in the morphological features (e.g., soma size, ramification, density, spacing) of the microglial cells of the paralaminar (PL) region of the amygdala in rhesus macaques raised with their biological mothers compared to individuals separated from their mothers 1 week or 1 month after birth. The manuscript was already reviewed for another journal and the authors responded adequately to the comments of the initial review. I have no major concerns with this manuscript.

Minor comments:

- Since there is no systematic measurement of stress, shouldn't "stress" be replaced with "mother separation" in the title? Or they should perhaps provide the critical details that previously showed that separation, even in the modified protocol, is indeed increasing physiological and behavioral markers. This is also important for the discussion, in which the data are interpreted in light of the stress induced by separation.

- Do you have photomicrographs illustrating the higher clusterization of microglial cells in 1-WS and 1-MS, compared to MR?

- Figure 6's legend could be a bit more detailed.
